# Deep Learning for Detecting Dental Plaque and Gingivitis From Oral Photographs: A Systematic Review

**DOI:** 10.1111/cdoe.70001

**Published:** 2025-06-26

**Authors:** Mohammad Moharrami, Elaheh Vahab, Mobina Bagherianlemraski, Ghazal Hemmati, Sonica Singhal, Carlos Quinonez, Falk Schwendicke, Michael Glogauer

**Affiliations:** ^1^ Faculty of Dentistry, University of Toronto Toronto Canada; ^2^ Department of Dental Oncology Princess Margaret Cancer Centre Toronto Canada; ^3^ Topic Group Dental Diagnostics and Digital Dentistry, ITU/WHO Focus Group AI on Health Geneva Switzerland; ^4^ Department of Periodontics and Oral Medicine School of Dentistry, University of Michigan Ann Arbor Michigan USA; ^5^ Department of Health Research Methods, Evidence, and Impact (HEI), McMaster University Hamilton Ontario Canada; ^6^ Dental Research Center Research Institute of Dental Science, Shahid Beheshti University of Medical Sciences Tehran Iran; ^7^ Health Promotion, Chronic Disease and Injury Prevention Department Public Health Ontario Toronto Canada; ^8^ Schulich School of Medicine & Dentistry, Western University London Canada; ^9^ Department of Conservative Dentistry and Periodontology LMU University Hospital Munich Germany; ^10^ Department of Dentistry, Centre for Advanced Dental Research and Care Mount Sinai Hospital Toronto Canada

**Keywords:** deep learning, dental plaque, gingivitis, RGB images, teledentistry

## Abstract

**Objectives:**

This systematic review aimed to evaluate the performance of deep learning (DL) models in detecting dental plaque and gingivitis from red, green, and blue (RGB) intraoral photographs.

**Methods:**

A comprehensive literature search was conducted across Medline, Scopus, Embase, and Web of Science databases up to January 31, 2025. The methodological characteristics and performance metrics of studies developing and validating DL models for classification, detection, or segmentation tasks were analysed. The risk of bias was assessed using the quality assessment of diagnostic accuracy studies 2 (QUADAS‐2) tool, and the certainty of the evidence was evaluated with the grading of recommendations assessment, development, and evaluation (GRADE) framework.

**Results:**

From 3307 identified records, 23 studies met the inclusion criteria. Of these, 10 focused on dental plaque, 11 on gingivitis, and two addressed both outcomes. The risk of bias was low in all QUADAS‐2 domains for 11 studies, with low applicability concerns in nine. For dental plaque, DL models showed robust performance in the segmentation task, with intersection over union (IoU) values ranging from 0.64 to 0.86 (median 0.74). Three studies indicated that DL models outperformed dentists in identifying dental plaque when disclosing agents were not used. For gingivitis, the models demonstrated potential but underperformed compared to dental plaque, with IoU values ranging from 0.43 to 0.72 (median 0.63). The certainty of the evidence was moderate for dental plaque and low for gingivitis.

**Conclusions:**

DL models demonstrate promising potential for detecting dental plaque and gingivitis from intraoral photographs, with superior performance in plaque detection. Leveraging accessible imaging devices such as smartphones, these models can enhance teledentistry and may facilitate early screening for periodontal disease. However, the lack of external testing, multicenter studies, and reporting consistency highlights the need for further research to ensure real‐world applicability.

## 1 Introduction

Dental plaque, a structured biofilm composed of bacteria, plays a pivotal role in the onset of gingivitis, which in turn can progress to periodontitis if left untreated [1]. This progression is significant, as periodontitis is not only a leading cause of tooth loss but is also associated with systemic health conditions such as cardiovascular disease and diabetes [1, 2]. The detection and early management of dental plaque and gingivitis are therefore crucial in preventing irreversible damage to the dentition and maintaining oral and systemic health [3, 4, 5]. Epidemiological studies from different countries show that periodontal disease can be prevalent but disproportionately affects vulnerable populations, such as low‐income individuals, older adults, and those living with a disability [1, 4, 6]. These populations may face barriers to accessing routine dental care, leading to delayed diagnosis [7]. Early screening initiatives may mitigate these disparities by identifying at‐risk individuals before the disease progresses, reducing the long‐term burdens.

With advancements in artificial intelligence (AI) and machine learning (ML), there is a growing interest in leveraging these technologies for early screening of oral health conditions [8]. Deep learning (DL) algorithms, a subset of ML, have shown great potential due to their end‐to‐end learning capabilities, automating both feature extraction and outcome prediction [9]. However, most DL applications in dentistry have been focused on assisting clinical diagnosis or decision‐making through data typically collected as part of routine care, such as radiographs [10, 11] or specialised forms of intraoral imaging, such as fluorescence imaging [12]. These imaging techniques, while valuable, are not accessible to the general public and often require clinical environments. In contrast, DL models using coloured red, green, and blue (RGB) intraoral photographs obtained from widely available devices such as smartphones are less resource intensive and demanding [13]. A combination of DL with such a readily available imaging modality, which has shown reliability for remote assessment [14, 15, 16], can further enhance teledentistry and democratise scalable solutions, making oral health screening feasible for a broader population.

The objective of this systematic review is to consolidate the evidence supporting the performance of DL models on RGB intraoral photographs, commonly obtained from routine sensors such as smartphones, intraoral cameras, and professional cameras (e.g., digital single‐lens reflex cameras), in detecting dental plaque and gingivitis. This review provides a comprehensive comparison of DL models across key computer vision tasks, including classification, which assigns a label to the entire image; detection, a combination of localisation and classification where the model draws boundary boxes around the object of interest; and segmentation, where each pixel is assigned a label, enabling precise contouring of objects [17].

## 2 Materials and Methods

### 2.1 Protocols and Registration

This systematic review was conducted and reported in accordance with the guidelines of the preferred reporting items for systematic reviews and meta‐analyses (PRISMA) statement (Appendix S1) [18, 19]. This review also adheres to the synthesis without meta‐analysis (SWiM) reporting guidelines, which complement PRISMA for systematic reviews where meta‐analysis is not feasible (Appendix S2) [20]. The research question was formulated using the participant, intervention, comparison, outcomes, time, and setting (PICO‐TS) criteria [21, 22, 23]. The study protocol was registered on the International Prospective Register of Systematic Reviews (PROSPERO) platform under the registration number CRD42024597073.

### 2.2 Search Strategy

Four electronic databases including Medline, Scopus, Embase, and Web of Science were used to identify publications that met the inclusion criteria. Besides the electronic databases, a snowball search was conducted by examining the reference lists of publications eligible for full‐text review and using Google Scholar to locate and assess studies that cited them. The search was conducted up to January 31, 2025, and the details of the search strategy, adapted for different databases, are shown in Appendix S3.

### 2.3 Eligibility Criteria

The following inclusion criteria were applied to screen and assess the retrieved records:

1. Original peer‐reviewed articles.
2. Observational diagnostics accuracy studies [24, 25] and if comparing different index tests (i.e., DL models) were applicable, a fully paired design [26].
3. Reports on DL models used for the classification, detection, or segmentation of dental plaque or gingivitis.
4. Models developed exclusively using intraoral photographs captured by sensors detecting visible wavelengths, including those from smartphones, intraoral cameras, or professional cameras.
5. Performance metrics of the DL models are reported separately for dental plaque or gingivitis.

Records were omitted if they satisfied any of the following conditions:

1. Animal studies.
2. Utilised traditional ML models or pipelines that required manual feature engineering and extraction, in contrast to end‐to‐end DL models such as convolutional neural networks (CNNs) or transformers, which perform automatic feature extraction.
3. Were based on images other than intraoral photographs, such as radiographs, infrared transillumination, fluorescence imaging, or spectral images.
4. Reported the performance metrics on combined or merged outcomes.
5. Reported on a validation of the model without including model development.

### 2.4 Focused PICO‐TS Question

Can DL models be used reliably to screen gingivitis and dental plaque from RGB intraoral photographs?

*Participants (P)*: Digital intraoral photographs, regardless of the participant's age.

*Intervention (I)*: Application of DL models for detecting gingivitis and dental plaque.

*Comparison (C)*: Annotated version of images following reference standards. These reference standards were established either through visual assessment of the original images by oral healthcare professionals or through clinical examinations and records.

*Outcomes (O)*: Performance metrics for computer vision tasks for dental plaque and gingivitis (detailed in Section 2.8).

*Timeframe (T)*: Cross‐sectional diagnostic accuracy studies, incorporating studies with either retrospective or prospective data collection [24, 25].

*Settings (S)*: Various clinical settings such as hospitals, private clinics, and academic institutes, as well as communities and non‐clinical settings.

### 2.5 Selection of Studies

A two‐stage screening (title‐abstract and full text) was carried out by three authors independently (GH, MB, and EV). Title management was conducted using commercially available software (Covidence systematic review software, Veritas Health Innovation, Melbourne, Australia). Duplicate records were eliminated both within each database and across databases. Full‐text versions of potentially relevant studies were retrieved and evaluated based on an eligibility form. Discrepancies in study selection were resolved through discussion, with the final decision made by the fourth author (MM). The reasons for excluding articles that did not meet the eligibility criteria were documented.

### 2.6 Data Extraction

Using a predesigned form, the following information was extracted from articles that met the eligibility criteria: title, authors' names, authors' affiliations, data source, year of publication, sample size, training‐validation‐testing ratio, frequency of cases, reference standard, ground truth measurement, type of dentition (permanent or primary), region of interest, image augmentation methods, image field of view, DL algorithms used, type of testing (internal vs. external), computer vision task (classification, detection, or segmentation), performance metrics, and authors' conclusions. For each included study, two of the three co‐authors (GH, MB, and EV) were randomly chosen to extract all relevant data independently. Any discrepancies between their extractions were then reviewed and resolved by the third co‐author (MM).

### 2.7 Risk of Bias

The quality assessment was conducted using the quality assessment of diagnostic accuracy studies (QUADAS‐2) criteria independently by three authors (GH, MB, and EV). The QUADAS‐2 checklist includes four domains that evaluate the risk of bias: patient selection, the index test, the reference standard, and flow and timing. Additionally, it includes three domains assessing applicability concerns related to patient selection, index test, and reference standard [27]. In this review, a modified version of QUADAS‐2, as detailed in prior studies [11, 13], was utilised. A domain's risk of bias was categorised as “low” if all signalling questions were answered “yes.” Conversely, if any signalling question was marked “no,” the risk was deemed “high.”

### 2.8 Data Analysis and Synthesis

Studies were grouped by the outcome of interest, either dental plaque or gingivitis, and by the computer vision task employed, which included classification, detection, or segmentation, as these groupings reflect the distinct performance metrics and clinical implications. Due to the differing nature of the computer vision tasks, a variety of metrics were reported. For classification tasks, the focus was mainly on the F1‐score due to imbalanced datasets, but metrics including recall (sensitivity), specificity, precision, accuracy, area under the receiver operating characteristic curve (AUROC), and area under the precision‐recall curve (AUPRC) were also considered, as detailed elsewhere [28]. For the localization component of the detection task, the focus was mean intersection over union (mIoU), which provides overall performance. For segmentation tasks, the Jaccard index (mIoU) was primarily used, but Dice score (F1‐score) and pixel accuracy were also considered. Where possible, metrics such as the F1‐score, which provides a harmonic mean of recall and precision, were calculated from available metrics.

Given the heterogeneity in study designs, computer vision tasks, metrics, and outcomes that precluded meta‐analysis, a narrative synthesis approach was adopted, qualitatively describing performance metrics and providing summary statistics (median and range). A threshold of at least three studies reporting the same metric for a given task and outcome was required, and all studies meeting inclusion criteria were included in the narrative synthesis, and the risk of bias was factored into the interpretation of findings. Heterogeneity was explored descriptively by assessing differences in study characteristics, such as the type of imaging sensor, reference standard, computer vision tasks, and DL algorithms. Potential reporting bias was assessed by comparing the range of reported performance metrics with expected outcomes based on study design and methodological standards. The certainty of evidence was evaluated using the grading of recommendations assessment, development, and evaluation (GRADE) framework for diagnostic test studies [29]. Finally, data were presented in tabular form, with separate tables detailing study characteristics and performance metrics for dental plaque and gingivitis, organised by computer vision task.

## 3 Results

### 3.1 Study Selection and Search Results

**FIGURE 1:**
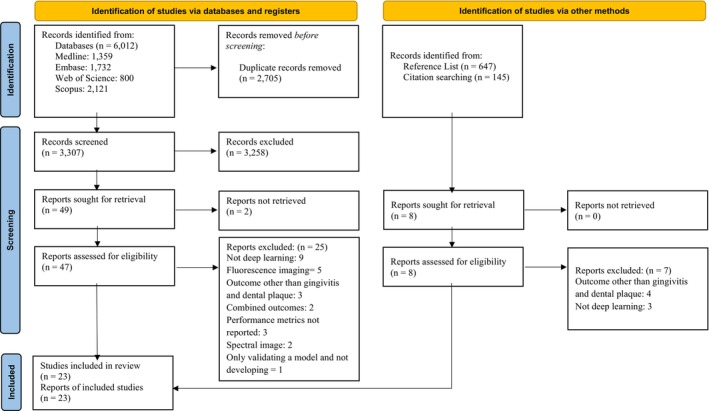
The flowchart of the search process based on the PRISMA 2020 statement.

The search resulted in 3307 unique records. After screening the titles and abstracts, 3258 records were excluded. From the remaining 49 records, two articles could not be retrieved [30, 31]. From the 47 retrieved records, 25 articles were excluded in the full‐text reading stage (Appendix S4): five articles used fluorescence imaging [32, 33, 34, 35, 36], three did not report on performance metrics [37, 38, 39], nine studies used either traditional ML or models other than DL [40, 41, 42, 43, 44, 45, 46, 47, 48], the outcome in three studies was not dental plaque or gingivitis [49, 50, 51], two articles were on combined outcomes not reporting metrics separately for gingivitis or dental plaque [52, 53], two articles reported on 3D and spectral imaging [54, 55], and one study did not develop a new model but instead focused on validating an existing one [56]. The remaining 22 studies were ultimately processed for data extraction [57, 58, 59, 60, 61, 62, 63, 64, 65, 66, 67, 68, 69, 70, 71, 72, 73, 74, 75, 76, 77, 78]. After manually screening Google Scholar for records that cited the included studies, one more study was added, bringing the total to 23 studies [79]. The search strategy is summarised in the PRISMA flowchart shown in Figure 1.

### 3.2 Study Characteristics

Overall, 12 articles reported on dental plaque detection (Table 1), with sample sizes ranging from 168 to 3932 images. Except for two studies, which collected data from a private clinic, the data for the remaining studies were sourced from either hospitals, academic institutions, or communities. The frequency of positive outcomes was reported in only one study. All studies utilised visual assessment to annotate the image to serve as the reference standard, with nine using disclosing agents. Six studies focused on images of permanent teeth, one on the primary dentition, and five on a mix of primary and permanent teeth. Five studies used professional cameras, two used intraoral cameras, three used smartphone cameras, and two studies employed a combination of professional and intraoral or smartphone cameras. Additionally, eight studies applied augmentation techniques to synthetically increase sample sizes. The field of view of the processed images fed into the models was a single tooth in seven studies and multiple teeth in five studies (Table 1).

**TABLE 1:** Characteristic and performance of included studies on the dental plaque outcome in chronological order.

First author (Year)	Sample size	Data source/Country	Training/Validation/Test	Distribution of cases	Classification system/classes	Reference standard/Annotators	Type of dentition (Primary, Permanent)	Tooth surface (Buccal, Occlusal, Lingual)	Image acquisition device	Image augmentation	Image field of view (Single vs. Multiple teeth)	Deep learning algorithms	Computer vision task	Best model performance metrics
Liang (2020)	Patients: 500 Images: 3182	Stomatology Hospital/China	Train: 2182 Test: 1000	712 out 3182 images	NM	Visual assessment of images/three dentists	Permanent	Buccal	Smartphone	Yes: Spatial shifts (random crops, rotations, and scaling), colour channel shifts (hue, saturation, exposure)	Multiple	Custom Multitask CNN	Classification	AUC: 0.74 AUC 1st (Sensitivity: 0.73, False Positives Rate: 0.46) AUC 2nd (Sensitivity: 0.56, False Positives Rate: 0.19)
Li (2020)	Images: 607	Pecking University School and Hospital/China	Train: 320 (children) Test: 287 (177 children and 110 adults)	NM	NM	Visual assessment of images using disclosing agents/Dentists (numbers NM)	Permanent and Primary	Buccal and lingual	Intraoral camera (Oral Endoscope)	NM	Single tooth (cropped out of original images)	Custom CNN Pipeline^a^ *Comparator*: DeepLabV3+	Segmentation	*DL for Children Data* IoU: 0.86 Accuracy: 0.86 *Dentist for Children Data* IoU: 0.69 *DL for Adult Data* IoU: 0.64
You (2020)	Patients: 86 Images: 886 by intraoral camera (Training and validation) Images: 98 by professional camera (testing)	Department of Paediatric dentistry, Peking University School and Hospital of Stomatology/China	Train: 80% (709) Validation: 20% (77) Test: 98	NM	NM	Visual assessment of images using disclosing agents/One Dentists	Primary	Buccal	Intraoral camera: TPC Ligang Professional camera: Canon EOS 60D	NM	Single tooth (cropped out of original images)	DeepLabV3+	Segmentation	*DL Model*: mIoU: 0.73 ± 0.17 *Dentist*: Initial Diagnosis (mIoU): 0.70 ± 0.26 After 1 Week (mIoU): 0.69 ± 0.25
Li (Wen) (2021)	Patients: 625 Images: 3932 (746 plaque)	Department of Periodontics, orthodontics and endodontics, Nanjing Stomatological Hospital, Nanjing University/China	Train: 424 Validation: 116 Test: 206	NM	NM	Visual assessment of images/three dentists	Permanent (adults)	Buccal	Smartphone and Professional Camera: iPhone 7, iPhone 8, Samsung Galaxy S8, Canon 6D	Yes: random shifts, crops, rotations, scaling, and colour channel shifts	Multiple	Custom Multi‐Task Learning CNN^a^ *Comparators*: VGG‐16 Residual‐50	Classification	AUC: 0.79 AUC 1st (Sensitivity: 0.78, Specificity: 0.59) AUC 2nd (Sensitivity: 0.56, Specificity: 0.80)
Andrade (2022)	Patient: 160 Image: 480	One private office/Brazil	Train: 360 Validation: 60 Test: 60	NM	NM	Visual assessment of images using disclosing agents/One dentist Validated by intra‐class correlation	Permanent and Primary	Buccal	Professional Camera: Canon EOS Rebel T3 camera	Yes: Horizontal flipping, random zoom, random rotation	Multiple	U‐Net with EfficientNet‐B4 as the backbone	Segmentation	(Pixelwise) Accuracy: 0.92 F1‐score (Dice): 0.61 Specificity: 0.94 Sensitivity: 0.67
Li (2022)	Images: 2884 (884 children, 2000 adults) Images: 15 new for test	Peking University School and Hospital of Stomatology/China	Train: 2020 Validation: 432 Test: 432	NM	NM	Visual assessment of images using disclosing agents/Dentists (numbers NM)	Primary and permanent teeth	Buccal and lingual based on camera	Intraoral Camera (oral endoscope)	NM	Single Tooth	FANet (custom feature‐fusion attention network)^a^ *Comparator*: DeeplabV3 DeeplabV3+ OCNet CCNet Unet++ others	Segmentation	*Internal Total*: Accuracy: 0.84 mIoU: 0.73 F1‐score (Dice): 0.84 *Internal Primary* mIoU: 0.73 *Internal permanent* mIoU: 0.65 *External*: Accuracy: 0.85 mIoU: 0.72
Shi (2023)	Images: 1304	Shanghai Shanda dental clinic/China	Train: 1043 Validation: 131 Test: 130	NM	NM	Visual assessment of images using disclosing agents/six people guided by professional dentists and re‐re‐checked by dentists	Permanent (adults)	Buccal, Occlusal, Lingual	Professional Camera: HD camera (Cannon 750D, Japan)	Yes: Horizontal and vertical flipping	Single tooth	Semantic decomposition network (SDNet)^a^ *Comparators*: Unet ResUNet Attention Unet MedT KiUnet TransUNet R2Unet Channel Unet	Segmentation	mIoU: 0.82 F1‐score (Dice): 0.83
Yüksel (2024)	Patients: 20 Images: 168	NM/Turkey	Train: 140 Test: 28	NM	NM	Visual assessment of images using disclosing agents/One dentist	Permanent	Buccal	Professional Camera (Canon EOS 850D)	NM	Single tooth	DeepLabV3+	Segmentation	*DL Model*: Accuracy: 0.87 Sensitivity: 0.84 Precision: 0.83 F1‐score (Dice): 0.83 Specificity: 0.89 mIoU: 0.76 mAP: 0.87 AUC: 0.92 *Dentist*: mIoU: 0.71 AUC: 0.83
Chen (2024)	Patients: 70 Images: 210	NM/China	Train: 80% Validation: 10% Test: 10%	NM	Quigley‐Hein Index (QHI) ranging from 0 to 5	Visual assessment of images using disclosing agents/Two dentists	Permanent (few deciduous)	Buccal	Professional Camera: Nikon D90	Yes: Horizontal and vertical flipping, random rotation	Single tooth (through the pipeline single tooth was obtained)	Custom pipeline: YOLOv8 detection and localization) + SAM (segmenting a tooth) + DeepPlaq (ResNeXt50 with channel and spatial attention)^a^ *Comparators*: Logistic regression VGG‐16 SqueezeNet ResNet18 ResNet50 ResNeXt50	Classification	Sensitivity: 0.77 Precision: 0.78 Accuracy: 0.77 F1‐Score: 0.77
Sobrinho (2024)	Patients: 160 Images: 480	One private clinic/Brazil	Train: 360 Validation: 60 (6‐fold CV) Test: 60	NM	NM	Visual assessment of images/One dentist Validated using an intra‐examiner agreement	Permeant and deciduous	Buccal	Professional Camera: Canon EOS Rebel T3	Yes: horizontal flip, random zoom, random rotation random crops	Multiple Teeth	‐UNet with EfficientNetB4 backbone using an ensemble strategy	Segmentation	(Pixelwise) Accuracy: 0.93 Sensitivity: 0.65 Specificity: 0.96 F1‐score (Dice): 0.63
Pedraza (2024)	Patients: 177 Images: 531	Communities in different regions/Mexico	Training: 431 Validation: 100	NM	NM	Visual assessment of images using disclosing agents/NM	Permeant and deciduous	Buccal	Smartphones	Yes: MixUp, CutMix	Multiple teeth	YOLOv11^a^ *Comparators*: YOLOv9 YOLOv10	Detection	Precision: 0.794 Sensitivity: 0.654 mAP@50: 0.713
Nantakeeratipat (2024)	Patients: 100 Images: 600	100 dental students from the Faculty of Dentistry at Srinakharinwirot University/Thailand	Three‐class model (images): Training: 240 Validation: 30 Test: 30 Two‐class model: Training: 240 Validation: 30 images Test: 29 images	NM	The percentage of the categorise plaque levels: mild: <30% dyed area moderate: 30%–60% Heavy: >60%	automated image‐analysis workflow using disclosing agents/NM	Permeant	Buccal	Smartphone	Yes: Flipping, rotation	Single tooth	Google Cloud's Vertex AI AutoML platform was used (specifically, the M125 release with Gemini 1.5 Pro)	Classification	Three‐class model AUPRC: 0.90 Precision: 0.86 Sensitivity: 0.83 F1‐score: 0.85 Two‐class model (acceptable vs. unacceptable) AUPRC: 0.96 Precision: 0.93 Sensitivity: 0.93 F1‐score: 0.93

Abbreviations: AUC, area under the receiver operating characteristic curve; CNN, convolutional neural network; DL, deep learning; IoU, intersection over union; mAP, mean average precision; NM, not mentioned.

^a^ Best performing model.

Thirteen articles reported on gingivitis outcomes (Table 2), with sample sizes ranging from 134 images to 7211 images. Most studies sourced data from hospitals or academic institutions, except for two using public repositories, one from a private clinic and another from an intraoral camera manufacturer. Six out of 13 articles reported the frequency of positive outcomes. To establish the annotated version of the images to serve as a reference standard, 10 studies relied on the original images, two studies relied on clinical examinations, and one study did not describe the process. Four studies used professional cameras, two used smartphones, three used intraoral cameras, and three employed a combination of smartphone and professional cameras to capture the images, and one study did not report. Nine out of 13 studies employed image augmentation techniques to synthetically increase sample sizes. For all studies, the field of view of the images fed into the DL models included multiple teeth (Table 2).

**TABLE 2:** Characteristic and performance of included studies on the gingivitis outcome in chronological order.

First author (Year)	Sample size	Data source/Country	Training/Validation/Test	Distribution of cases	Classification system/Classes	Reference standard/Annotators	Type of dentition (Primary, Permanent)	Tooth surface (Buccal, Occlusal, Lingual)	Image acquisition device	Image augmentation	Image field of view (Single vs. Multiple teeth)	Deep learning algorithms	Computer vision task	Best model performance metrics
Alalharith (2020)	Patients: 47 Images: 134 Regions: 804	College of Dentistry, Imam Abdulrahman Bin Faisal University/Saudi Arabia	Train: 80% (107) Test: 20% (27)	Inflamed: 37.9% (305) Non‐inflamed: 62.1% (499)	Yes/Inflamed non‐inflamed	Visual assessment of images/dentists (numbers NM)	NM	Buccal	Intraoral Camera	NM	Multiple	Faster R‐CNN with ResNet‐50 as a feature extractor	Detection	Accuracy: 0.77 Precision: 0.88 Sensitivity: 0.42 F1‐score: 0.57 (manual) mAP: 0.68
Carillo (2020)	Patients: 50 Images: 200	One private office/Philippines	Train: 200 Test: 200 (internet)	Healthy gums: 50 Gingivitis: 50 Periodontitis: 50 Advanced periodontitis: 50	Yes/healthy gingivitis periodontitis sever disease	Clinical examination/Four dentists	NM	Buccal	Smartphone	Yes: rotations, height and width shift	Multiple	Custom CNN with 6 convolution layers and 1 fully connected layer	Classification	Accuracy: 0.83
Liang (2020)	Patients: 500 Images: 3182	Stomatology Hospital/China	Train: 2182 Test: 1000	Gingivitis (positive): 1744 out 3182 images	NM	Visual assessment of images/three dentists	Permanent	Buccal	Smartphone	Yes: Spatial shifts (random crops, rotations, and scaling), colour channel shifts (hue, saturation, exposure)	Multiple	Custom Multitask CNN	Detection	*Classification*: AUC: 0.79 AUC 1st (Sensitivity: 0.78, False Positives Rate: 0.32) AUC 2nd: (Sensitivity: 0.68, False Positives Rate: 0.14) *Localization*: FROC 1st (Sensitivity: 0.51, False Positives per Image: 3.7) FROC 2nd (Sensitivity: 0.34. False Positives per Image: 1.3)
Li (2021)	Patients: 110 Images: 447	Faculty of Dentistry, The University of Hong Kong/Hong Kong	Train: 337 Test: 110	NM	NM/ 0. background 1. diseased 2. healthy 3. questionable diseased 4. questionable healthy	Visual assessment of images/one dentist	NM	Buccal	Professional Camera: SLR camera (EOS 500D Canon)	Yes: random crop, rotation, and vertical flip	Multiple	DeepLabv3+ (backbone: MobileNetV2, Xception65)	Segmentation	2‐Class Segmentation: AUC: 0.70 Precision: 0.60 Sensitivity: 0.41 mIoU: 0.65 5‐Class Segmentation: Xception65—mIoU: 0.37 MobileNetV2—mIoU: 0.35 4‐Class Segmentation: Xception65—mIoU: 0.49 MobileNetV2—mIoU: 0.45
Shang (2021)	Images: 7211	Zhuhai AICREATE Medical Technology Co., LTD/China	Trani: 6489 Test: 722	NM	NM	Visual assessment of images/ (numbers NM)	NM	Buccal, Occlusal, Lingual	Intraoral Camera	NM	Multiple	U‐Net with VGG16 as the backbone^a^ *Comparators* DeepLabV3 PSPNet	Segmentation	mIoU: 0.43 mAP: 0.55
Li (wen) (2021)	Patients: 625 Images: 3932 (3175 gingivitis)	Department of Periodontics, orthodontics and endodontics, Nanjing Stomatological Hospital, Nanjing University/China	Train: 1726 Validation: 469 Test: 980	NM	Yes	Visual assessment of images/Three dentists	permanent (adults)	Buccal	Smartphone and Professional Cameras: iPhone 7, iPhone 8, Samsung Galaxy S8, Canon 6D	Yes: random shifts, crops, rotations, scaling, and colour channel shifts	Multiple	Custom Multi‐Task Learning CNN^a^ *Comparators*: VGG‐16 Residual‐50	Detection	*Classification*: AUC:0.87 AUC 1st (Sensitivity: 0.88, Specificity: 0.63) AUC 2nd (Sensitivity: 0.60, Specificity: 0.83) *Localization*: FROC 1st: 0.58 (Sensitivity: 0.66, False Positives per Image: 1.5) FROC 2nd: 0.58 (Sensitivity: 0.43, False Positives per Image: 0.58)
Kurt‐Bayrakdar (2022)	Images: 654 (519 gingivitis)	Eskişehir Osmangazi University, Faculty of Dentistry, Department of Orthodontics/Turkey	Train: 80% (417) Validation: 10% (51) Test: 10% (51)	NM	Yes 1. mild 2. moderate 3. sever	Visual assessment of images/Three periodontists	permanent (adults)	Buccal	Professional camera	NM	Multiple	YOLOv5x	Detection	AUC: 0.82 Sensitivity: 0.73 Precision: 0.82 F1‐score: 0.77
Chau (2023)	Images: 567	Comprehensive Dental Clinic of the University Dental Hospital of University of Hong Kong/China	Train 80% (453 images) Validation 20% (114)	Train (#pixels) Healthy: 9270413 Diseased: 5711027 Questionable: 4596612 Test (#pixels) Healthy: 1579914 Diseased: 1604543 Questionable: 1477867	1. Healthy 2. questionable 3. diseased	Visual assessment of images/one calibrated dentist	Permanent (adults)	Buccal	Professional camera: Canon EOS 700D	Yes: Cropping, rotating, flipping	Multiple	DeepLabV3	Segmentation	mIoU: 0.60 Sensitivity: 0.92 Specificity: 0.94
Li (2024)	Patients: 134 Images: 683	Department of Periodontics, Orthodontics and Endodontics, Stomatological Hospital/China	*Gingivitis* Train: 66.7% Validation 16.7% Test: 16.7% *Control* Train: 66.3% Validation: 16.9% Test: 16.9%	Gingivitis: 600 Healthy: 83	NM	Clinical examination/post‐graduate dentists (numbers NM)	Permanent (adult)	Buccal	Smartphone and Professional camera: iPhone, Samsung Galaxy, Canon 6D	Yes: random rotations, zooming, and horizontal flipping	Multiple	GoogLeNet^a^ ResNet^a^ *Comparators*: AlexNet VGG	Classification	*ResNet*: Precision: 0.97 Sensitivity: 0.87 F1 Score: 0.92 Cross‐Entropy Loss: 0.38 Accuracy: 0.87 AUC: 0.97 *GoogleNet*: Precision: 0.98 Sensitivity: 0.91 F1 Score: 0.93 Cross‐Entropy Loss: 0.32 Accuracy: 0.90 AUC: 0.94
Wen (2024)	Images: 826	School and Hospital of Stomatology, Wuhan University/China	Train: 80% Test: 20%	Grade 0: 152 Grades I, II, III, IV: 674	Modified Gingival Index/Grade 0 to IV	Visual assessment of images/Three out of six dental specialists after training with help of clinal history and records	Permanent (adult)	Buccal	Professional Camera: Canon EOS Rebel T3	NM	Multiple	DensNet (plus U‐Net for segmenting teeth)^a^ *Comparators*: ResNet Inception‐3 EffcinetNet	Segmentation Classification	Segmentation mIoU (model): 0.72 mIoU (two dentists): ~0.68 Classification AUC: 0.82 Sensitivity: 0.80 Specificity: 0.68 Accuracy: 0.76
Cheng (2024)	Images: 415	Faculty of Dentistry, The University of Hong Kong/China	Training: 250 Validation: 82 Test: 83	NM	1. diseased 2. healthy 3. questionable, 4. background	Visual assessment of images/Two dentists	permanent	Buccal	Professional cameral: Canon EOS 500D Smartphone Camera	Yes: random cropping, random rotation, vertical flipping	Multiple	Semantic segmentation network based on DeepLabv3+ Backbone: ResNet50	Segmentation	Sensitivity: 0.94 Specificity: 0.99 mIoU: 0.51
Liu (2024)	Images: 3365	Publicly available images (e.g., Kaggle)/unknown	Training: 2019 Validatio673 Test: 673	NM	NM	Visual assessment of images/Dentists (number unknown) using Segment Anything Model (SAM)	NM	Buccal, Occlusal, Lingual	Intraoral camera	Yes: random cropping, rotation, vertical flipping	Multiple	Oral‐Mamba^a^ ‐Modified Y‐net for localised features—Integrates a “Mamba block” in the bottleneck, which uses Selective SSM techniques Comparator: U‐net	Segmentation	IoU: 0.70 Sensitivity: 0.83 Precision: 0.82 Accuracy: 0.83 F1‐score (manual): 0.82
Ragadio (2024)	Images: 666	Kaggle dataset/unknown	Train: 70% Validation: 20% Test: 10%	NM	NM	NM	NM	NM	NM	Yes: rotations, shifts, shears, zooms, flips, brightness adjustments, and channel shifts	Multiple	Modified DenseNet121 with Transfer Learning	Classification	Precision: 0.95 Sensitivity: 0.98 F1‐score: 0.96

Abbreviations: AUC, area under the receiver operating characteristic curve; CNN, convolutional neural network; DL, deep learning; FROC, free‐response ROC; IoU, intersection over union; mAP, mean average precision; NM, not mentioned.

^a^ Best performing model.

### 3.3 Assessment of Methodological Quality

**FIGURE 2:**
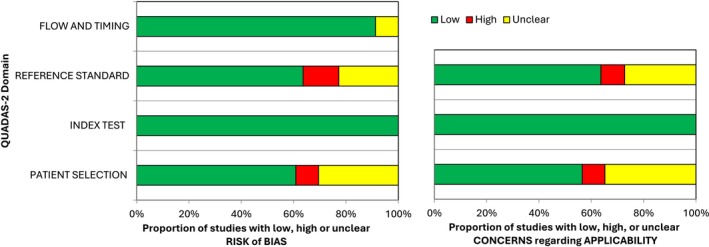
The proportion of studies with low, high, and unclear risk of bias or applicability concerns based on the QUADS‐2 tool.

Detailed information on the risk of bias and applicability concerns is presented in Appendix S5 and Figure 2. In terms of risk of bias, 11 studies were considered to have a low risk across all four QUADAS‐2 domains. For applicability concerns, nine studies were categorised as having low concern across all three domains. Regarding the domain‐specific risk of bias in patient selection, seven studies were rated as unclear and two as high, primarily due to non‐consecutive sample selection or restrictions to specific cohorts without describing the outcome distribution. The remaining studies were assessed as having a low risk of bias. For corresponding applicability concerns, nine studies were considered unclear, and two were rated as high, mostly due to small sample sizes or data drawn from a single private clinic. In terms of the reference standard, three studies had a high risk of bias, and five were unclear due to having only one annotator and a lack of calibration or insufficient description of the annotation process; the remaining studies were considered low risk. For the index test, all studies had a low risk of bias. Lastly, regarding flow and timing, two studies had an unclear risk of bias due to ambiguity concerning the consistent application of the reference standard to both the training and test sets, while the rest were rated as low risk.

### 3.4 Synthesis and Model Performance

Regarding dental plaque, seven studies focused on *segmentation tasks*, with mIoU appearing in five studies, ranging from 0.64 to 0.86, with a median of 0.74 [59, 60, 62, 66, 67]. Additionally, five studies also reported Dice‐scores (F1‐scores) ranging from 0.61 and 0.84 with a median of 0.83 [58, 59, 62, 66, 77]. Five studies also reported accuracy, with values ranging from 0.84 to 0.93 and a median of 0.87 [58, 59, 66, 67, 77]. Furthermore, four studies addressed *classification tasks*, with two reporting sensitivity based on the highest optimal point of the AUROC curve, at 0.73 and 0.78 [63, 64], while the others reported a sensitivity of 0.77 and 0.83 based on a default threshold [71, 74]. Notably, only one study reported on the *detection task* (Table 1).

In relation to gingivitis outcomes, six studies addressed *segmentation tasks*, with mIoU values ranging from 0.43 to 0.72 and a median of 0.63 [61, 63, 69, 72, 78, 79]. Four studies examined *detection*, and three of these studies provided AUROC values for the classification component of the detection task, ranging from 0.79 to 0.87 with a median of 0.82 [64, 68, 70]. Also, two other studies provided F1‐scores of 0.57 and 0.77 [57, 70]. Moreover, three studies focused on *classification tasks*, and two reported the F1‐scores of 0.92 and 0.96 [65, 75] (Table 2).

The potential for reporting bias due to missing results was assessed by examining the range of reported metrics. No substantial discrepancies were found between the study designs and reported results, except for one study that reported only accuracy [73], a metric influenced by disease prevalence and dataset imbalance, which is prone to overestimation. The remaining studies employed a diverse set of metrics aligned with their specific methodologies and computer vision tasks, which limited comprehensive synthesis since performance across different tasks is not directly comparable. Within each task, inconsistencies emerged based on each study's focus. For example, in segmentation analyses for dental plaque, five of seven studies reported mIoU to emphasise spatial overlap [59, 60, 62, 66, 67], while the other two studies only concentrated on pixel classification performance [58, 77]. These expected variations reflect differences in study objectives, ranging from clinical applicability to algorithmic innovation, rather than reporting bias.

The certainty of the evidence was assessed across five GRADE domains: risk of bias, indirectness, inconsistency, imprecision, and publication bias. Each domain was rated as either “no concern” (no downgrade) or “serious concern” (downgrade by one level) [29, 80]. For dental plaque detection, only the inconsistency domain was rated as a serious concern, resulting in an overall moderate certainty of the evidence. For gingivitis, serious concerns in the domains of indirectness and inconsistency led to two downgrades and an overall low certainty of the evidence (Table 3).

**TABLE 3:** The certainty of evidence assessment for included studies in the systematic review using grading of recommendations assessment, development, and evaluation (GRADE).

Outcome	No. of studies	Study design	Risk of bias	Indirectness	Inconsistency	Imprecision	Publication bias	Certainty of the body of evidence
Detecting dental plaque	12	Observational diagnostic accuracy study	Not serious	Not serious	Serious^b^	Not serious	Not serious	⊕ ⊕ ⊕⊙ Moderate
Detecting gingivitis	13	Observational diagnostic accuracy study	Not serious	Serious^a^	Serious^b^	Not serious	Not serious	⊕ ⊕ ⊙⊙ Low

^a^ The annotation of images, which served as the reference standard, was primarily based on the visual assessment of inflammation signs. This approach may be useful for epidemiological surveys and screening purposes. However, a definitive diagnosis relies on bleeding on probing, a criterion that was missing in many studies.

^b^ Inconsistencies due to the use of different deep learning algorithms, computer vision tasks, and evaluation metrics.

## 4 Discussion

This systematic review evaluated the performance of DL models in detecting dental plaque and gingivitis, two risk indicators for periodontal disease, utilising RGB intraoral photographs. To the authors' knowledge, this is the first comprehensive review to examine the application of DL models for detecting these outcomes across various computer vision tasks and DL algorithms using intraoral photographs. Despite some methodological heterogeneity, differences in DL pipelines, use of diverse imaging sensors (i.e., smartphones, intraoral cameras, and professional cameras) as well as inconsistencies in reporting metrics (which led to a downgrade of the GRADE inconsistency domain) DL models showed promising potential, particularly in detecting dental plaque from intraoral photographs.

Seven of the 12 dental plaque studies employed segmentation followed by classification and detection, compared to six of the 13 gingivitis studies that performed segmentation followed by detection and classification. Segmentation, by delineating the region of interest, offers more detailed information than detection (which relies on bounding boxes) or classification (which labels the entire image) [17]. Overall, models for dental plaque segmentation performed superior, with a median mIoU of 0.74 (range: 0.64–0.86), compared to a median mIoU of 0.63 (range: 0.43–0.72) for gingivitis. This difference may partly be attributed to the reference standard used in dental plaque studies, where disclosing agents were applied in nine studies, including all seven employing segmentations. This clear demarcation may facilitate more accurate annotation and segmentation of plaque [81, 82]. Moreover, while the field of view in gingivitis studies included multiple teeth and surrounding soft tissue, seven of the 12 dental plaque studies, including all five that reported mIoU for segmentation, used single‐tooth images obtained through preprocessing, which minimised background noise and isolated plaque features, thereby reducing visual complexity. Finally, the interaction between the DL algorithms and the outcome might also influence performance, warranting further investigation in future studies.

Considering the standards for reporting diagnostic accuracy (STARD) definition of a clinical reference standard, “The best available method for establishing the presence or absence of the target condition,” [25] the dental plaque studies in this review largely adhered to this definition. Visual assessment of dental plaque using disclosing agents is widely recognised as a reliable method in the literature [81, 82, 83, 84, 85, 86] and was adopted in 9 out of the 12 studies reviewed. In contrast, although clinical signs of inflammation, such as erythema and swelling can aid in the diagnosis of gingivitis, the definitive diagnosis relies on the criterion set by the 2017 World Workshop on the Classification of Periodontal and Peri‐Implant Diseases and Conditions, which defines gingivitis as the presence of bleeding on light probing at ≥ 10% of sites in the absence of probing attachment loss [85, 87]. This distinction is reflected in the GRADE scoring, which rated the indirectness domain as serious for gingivitis. Nonetheless, this review primarily aimed to determine whether DL models applied to oral photographs can be used for population‐level epidemiological screening, allowing high‐risk individuals to follow up with a clinician, and therefore, the results are applicable.

Among the sensors, professional cameras were used the most, followed by smartphones and intraoral cameras. Due to the lack of comparative studies, where the same photo is taken using different sensors and the same DL model is applied, it is not possible to determine whether the sensor type impacts model performance. However, in general, no noticeable trend in model performance was observed based on the sensor type, unlike findings from previous studies on dental caries [13]. This may be because most images were taken from labial and buccal views, rather than occlusal or lingual views, where intraoral cameras could offer superior access, magnification, and lighting. It should also be mentioned that some studies used different sensors for training and testing to enhance model generalisability. For instance, one study trained the model using images from intraoral cameras and tested it on images from professional cameras [60]. Another study explored this further by using two different types of intraoral cameras for training and testing [66].

Three comparative studies evaluated the accuracy of DL models against dentists for dental plaque detection, and based on the mIoU metric, all found the DL models to be more accurate than dentists when making diagnoses without the aid of disclosing agents: (0.76 vs. 0.71) [59], (0.73 vs. 0.70) [60], and (0.86 vs. 0.69) [67]. This superior performance can be attributed to DL models' ability to capture subtle, low‐contrast patterns in dental plaque that are difficult for the human eye to detect. Additionally, DL models maintain consistent performance without fatigue, are trained on large datasets, and provide standardised detection, eliminating human variability. As a result, these DL models may have the potential to replace disclosing agents, which temporarily stain the teeth, requiring subsequent cleaning. In addition, if deployed successfully, DL models may enhance patient motivation by providing personalised feedback and real‐time plaque detection, making oral hygiene management more convenient and engaging.

Moreover, two studies from the same team compared the performance of DL models between primary and permanent teeth in children and adults. One study trained the model on a children's dataset and tested it on a separate children's dataset, achieving a mIoU of 0.86, while testing on adults yielded a mIoU of 0.64 [67]. Another study also showed superior performance for primary teeth, with a mIoU of 0.73, compared to permanent teeth, with a mIoU of 0.65 [66], when trained on a mix of primary and permanent images; in this study, primary teeth showed a larger and more distinct plaque proportion. This makes the plaque features easier for the model to capture and segment. In contrast, permanent teeth tended to have smaller and less pronounced plaque patterns, which may have led to lower performance when the same model was applied [66]. Further comparative studies are needed to fully evaluate the performance of DL models between primary and permanent teeth.

Comparative studies have also shown that custom DL pipeline algorithms might outperform off‐the‐shelf models due to their ability to incorporate task‐specific adaptations. Li et al. enhanced segmentation accuracy by employing self‐attention mechanisms and multi‐scale feature fusion, allowing the algorithm to better capture the low‐contrast nature of dental plaque and improve precision in distinguishing plaque from surrounding teeth [66]. Shi et al. proposed a semantic decomposition network with contrastive and structural constraints, specifically addressing the challenges of semantic‐blur regions and variable plaque morphology [62]. Chen et al. further demonstrated that the integration of attention‐based mechanisms and tailored multi‐view intraoral image processing enhances the accuracy of plaque classification, as these techniques focus on relevant regions and minimise background noise [71]. Similarly, Liu et al. introduced Oral‐Mamba, a U‐Net variant that fuses local and global features via selective state space models, which improved segmentation for gingivitis [78].

In developing DL models, many studies struggled with small sample sizes and therefore performed data augmentation, which synthetically increases sample size, thereby decreasing overfitting and enhancing model generalisation through diverse training examples, which is vital given the challenges of acquiring large medical imaging datasets [88, 89]. However, augmented data may not fully capture the nuanced variability of real‐world intraoral images, and excessive or irrelevant transformations may introduce artefacts that obscure diagnostic features. Furthermore, if a model is underfitting, demonstrated by consistently high bias on the training set, then increasing the sample size is unlikely to improve performance [90, 91]. Additionally, as seen in most of the studies, the algorithms were not developed from scratch. While DL typically requires large datasets, techniques such as transfer learning, using pre‐trained data on a large general dataset and fine‐tuning it for a specific task, and data augmentation enabled effective model training with smaller sample sizes [92]. While these strategies can mitigate the limitations of small datasets, the ultimate goal should be to assemble larger and more diverse datasets, which can foster robust feature learning, especially in unbalanced datasets, and minimise the risk of overfitting and noise amplification, thereby improving performance on unseen data [93].

This review's narrative synthesis, limited by diverse computer vision tasks and metrics, may reduce the precision of conclusions as data cannot be quantitatively pooled. Additionally, grouping studies by outcome and task may miss other heterogeneity, potentially weakening the findings' robustness. Despite the synthesis limitation, this review showed that by leveraging RGB oral photographs and advanced image analysis, screening of dental plaque and gingivitis is feasible, which can be particularly important in underserved populations with limited access to dental care. Early detection may not only reduce the incidence of periodontal diseases but can also amplify secondary prevention efforts. Despite the promising performance of DL models, several limitations warrant consideration. Most studies were conducted in single‐centre settings, typically within a single hospital, academic institution, or private clinic, which limited their generalisability to broader populations and settings. Also, external testing was notably lacking. Moreover, although AI‐specific guidelines such as STARD‐AI are still under development [94], existing frameworks such as the 2015 version of STARD [25], the Checklist for Artificial Intelligence in Medical Imaging (CLAIM) [95], or the specific checklist developed for dental research [96] could have been adapted to improve transparency and reporting consistency.

## 5 Conclusions

DL models demonstrate promising potential for detecting dental plaque and gingivitis from intraoral photographs, with superior performance in plaque detection. When applied to images without disclosing agents, the models detected nuanced plaque features more effectively than dentists relying solely on unaided visual inspection. This highlights the potential of DL models to enhance teledentistry by utilising widely accessible RGB imaging devices, such as smartphones. However, the lack of external testing across diverse populations emphasises the need for further research to ensure model generalizability and real‐world applicability. Future studies should also focus on multicenter trials, standardised reporting, and assessing the cost‐effectiveness of these models, which will be essential for translating these promising findings into practical solutions for oral health care.

## Conflicts of Interest

The authors declare no conflicts of interest.

## Supporting information


Appendix S1

Appendix S2

Appendix S3

Appendix S4

Appendix S5

## Data Availability

Data sharing is not applicable to this article as no new data were created or analyzed in this study.
