# Supplementary material for: Deep Learning for Detecting Dental Plaque and Gingivitis From Oral Photographs: A Systematic Review
Source: Community Dent Oral Epidemiol. 2025 Jun 26;53(6):617–32. doi: 10.1111/cdoe.70001 (PMC12627268; doi:10.1111/cdoe.70001)
Supplement: Supplementary file 3 — Appendix S3 [file CDOE-53-617-s005.docx]

| **Appendix 3. Search query structures adapted for different databases** | | |
| --- | --- | --- |
| **Database** | **Field/Syntax** | **Query Structure** |
| **OVID Medline** | .mp (multi‑purpose) | **(Concept 1) AND (Concept 2) AND (Concept 3)**  • **Concept 1:** detect* OR segment* OR classif* OR recognize OR recognition OR diagnos* OR differentiat* OR discriminat* OR risk OR mark* OR "computer‑assisted" OR "image analysis" OR "pattern recognition"  • **Concept 2:** gingiva OR gingivitis OR "dental plaque" OR "tooth plaque" OR "teeth plaque" OR "dental biofilm" OR "tooth biofilm" OR "teeth biofilm" OR "periodontal disease" OR periodontitis OR "gum disease" OR "periodontal inflammation" OR "gum inflammation" OR "periodontal pocket" OR "periodontal attachment loss" OR "alveolar bone loss" OR "dental calculus" OR "tooth calculus" OR "teeth calculus" OR tartar OR "gum recession" OR "gingival recession"  • **Concept 3:** AI OR "artificial intelligence" OR "machine learning" OR "deep learning" OR "neural network" OR "supervised learning" OR "semi‑supervised learning" OR "unsupervised learning" OR "computer vision" OR "multilayer perceptron" OR MLP OR transformer OR "convolutional neural network" OR CNN OR "image processing" OR DL OR "vision model" OR LVM OR LLM |
| **OVID Embase** | .mp (multi‑purpose) | **(Concept 1) AND (Concept 2) AND (Concept 3)** Uses the same three concept groups as Medline, with .mp covering title, abstract, and indexing terms (e.g., Emtree). |
| **Scopus** | TITLE‑ABS‑KEY() | **TITLE‑ABS‑KEY( (Concept 1) AND (Concept 2) AND (Concept 3) )** Searches titles, abstracts, and keywords using the same keyword groups. |
| **Web of Science** | Topic=()  Searches for title, abstract, keywords plus, and authors’ keywords | **Topic=( (Concept 1) AND (Concept 2) AND (Concept 3) )** Searches topics (titles, abstracts, and keywords) with the identical keyword groups. |
