# Supplementary material for: Deep Learning for Detecting Dental Plaque and Gingivitis From Oral Photographs: A Systematic Review
Source: Community Dent Oral Epidemiol. 2025 Jun 26;53(6):617–32. doi: 10.1111/cdoe.70001 (PMC12627268; doi:10.1111/cdoe.70001)
Supplement: Supplementary file 4 — Appendix S4 [file CDOE-53-617-s002.docx]

| **Appendix 4.** Reasons for exclusion of studies at the full text reading a stage | | |
| --- | --- | --- |
| **Reference Number** | **First Author (Year)** | **Reason for Exclusion** |
| 1 | Rana A (2017) | Used fluorescence imaging |
| 2 | Yauney G (2019) |  |
| 3 | Wang C (2020) |  |
| 4 | Vila-Blanco N (2020) |  |
| 5 | Imangaliyev S (2016) |  |
| 6 | Rosini S (2023) | Did not report on performance metrics |
| 7 | Liu C (2022) |  |
| 8 | Lee J-Y (2024) |  |
| 9 | Ji Z (2010) | Used traditional machine learning or non-deep learning models requiring manual feature extraction |
| 10 | Kang J (2006) |  |
| 11 | Li W (2020) |  |
| 12 | Li W (2019) |  |
| 13 | Khaleel BI (2021) |  |
| 14 | Chen Y (2020) |  |
| 15 | Askarian B (2019) |  |
| 16 | Arabi PM (2018) |  |
| 17 | Coy E (2024) |  |
| 18 | Joo J (2019) | Outcome was not dental plaque or gingivitis |
| 19 | Alam MK (2024) |  |
| 20 | Moriyama Y (2019) |  |
| 21 | Ma T (2022) | Combined outcomes not reporting metrics separately for each outcome |
| 22 | Park S (2023) |  |
| 23 | Hossam A (2021) | Reported on 3D or spectral imaging |
| 24 | Li W (2025) |  |
| 25 | Chau RCW (2025) | Did not develop a model but validated an already developed one |

**References**

[1] Rana A, Yauney G, Wong LC, Gupta O, Muftu A, Shah P. Automated segmentation of gingival diseases from oral images. 2017 IEEE Healthcare Innovations and Point of Care Technologies (HI-POCT), IEEE; 2017, p. 144–7.

[2] Yauney G, Rana A, Wong LC, Javia P, Muftu A, Shah P. Automated process incorporating machine learning segmentation and correlation of oral diseases with systemic health. 2019 41st annual international conference of the IEEE engineering in medicine and biology society (EMBC), IEEE; 2019, p. 3387–93.

[3] Wang C, Qin H, Lai G, Zheng G, Xiang H, Wang J, et al. Automated classification of dual channel dental imaging of auto-fluorescence and white lightby convolutional neural networks. J Innov Opt Health Sci 2020;13:2050014.

[4] Vila-Blanco N, Freire V, Balsa-Castro C, Tomás I, Carreira MJ. DenTiUS plaque, a web-based application for the quantification of bacterial plaque: development and usability study. J Med Internet Res 2020;22:e18570.

[5] Imangaliyev S, van der Veen MH, Volgenant CMC, Keijser BJF, Crielaard W, Levin E. Deep learning for classification of dental plaque images. Machine Learning, Optimization, and Big Data: Second International Workshop, MOD 2016, Volterra, Italy, August 26-29, 2016, Revised Selected Papers 2, Springer; 2016, p. 407–10.

[6] Rosini S, Altamura S, Pietropaoli D, Placidi G, Polsinelli M. Periodontisis evaluation through automatic teeth detection and segmentation from self-collected smartphone images. 2023 IEEE 36th International Symposium on Computer-Based Medical Systems (CBMS), IEEE; 2023, p. 738–41.

[7] Liu C, Wu HP. Distribution analysis of dental plaque based on deep learning. 2022 IEEE International Conference on Consumer Electronics-Taiwan, IEEE; 2022, p. 185–6.

[8] Lee J-Y, Lim J-N, Han B-H, Seok S-H, Yoo H-J. Development of AI web service for quantification of dental plaque. International Journal of Clinical Preventive Dentistry 2024;20:27–32.

[9] Ji Z, Gong C. Segmentation and quantification of dental plaque using modified kernelized fuzzy C-means clustering algorithm. 2010 Chinese Control and Decision Conference, IEEE; 2010, p. 788–91.

[10] Kang J, Li X, Luan Q, Liu J, Min L. Dental plaque quantification using cellular neural network-based image segmentation. Intelligent Computing in Signal Processing and Pattern Recognition: International Conference on Intelligent Computing, ICIC 2006 Kunming, China, August 16–19, 2006, Springer; 2006, p. 797–802.

[11] Li W, Jiang X, Sun W, Wang S, Liu C, Zhang X, et al. Gingivitis identification via multichannel gray‐level co‐occurrence matrix and particle swarm optimization neural network. Int J Imaging Syst Technol 2020;30:401–11.

[12] Li W, Chen Y, Sun W, Brown M, Zhang X, Wang S, et al. Expression of Concern: A gingivitis identification method based on contrast‐limited adaptive histogram equalization, gray‐level co‐occurrence matrix, and extreme learning machine. Int J Imaging Syst Technol 2019;29:77–82.

[13] Khaleel BI, Aziz MS. Using artificial intelligence methods for diagnosis of gingivitis diseases. J Phys Conf Ser, vol. 1897, IOP Publishing; 2021, p. 012027.

[14] Chen Y, Chen X. Gingivitis identification via GLCM and artificial neural network. Medical Imaging and Computer-Aided Diagnosis: Proceeding of 2020 International Conference on Medical Imaging and Computer-Aided Diagnosis (MICAD 2020), Springer; 2020, p. 95–106.

[15] Askarian B, Tabei F, Tipton GA, Chong JW. Smartphone-based method for detecting periodontal disease. 2019 IEEE Healthcare Innovations and Point of Care Technologies,(HI-POCT), IEEE; 2019, p. 53–5.

[16] Arabi PM, Naveen TS, Vamsha Deepa N, Samanta D. Automatic diagnosis of dental diseases. Smart and Innovative Trends in Next Generation Computing Technologies: Third International Conference, NGCT 2017, Dehradun, India, October 30-31, 2017, Revised Selected Papers, Part I 3, Springer; 2018, p. 363–75.

[17] Coy E, Santo W, Jue B, Betts H, Ramos-Gomez F, Gansky SA. Among artificial intelligence/machine learning methods, automated gradient-boosting models accurately score intraoral plaque in non-standardized images. J Calif Dent Assoc 2024;52:2422146.

[18] Joo J, Jeong S, Jin H, Lee U, Yoon JY, Kim SC. Periodontal disease detection using convolutional neural networks. 2019 International Conference on Artificial Intelligence in Information and Communication (ICAIIC), IEEE; 2019, p. 360–2.

[19] Alam MK, Alanazi NH, Alshehri ADA, Chowdhury F. Accuracy of Al algorithms in diagnosing periodontal disease using intraoral images. J Pharm Bioallied Sci 2024;16:S583–5.

[20] Moriyama Y, Lee C, Date S, Kashiwagi Y, Narukawa Y, Nozaki K, et al. A map reduce-like deep learning model for the depth estimation of periodontal pockets. HEALTHINF, 2019, p. 388–95.

[21] Ma T, Zhou X, Yang J, Meng B, Qian J, Zhang J, et al. Dental lesion segmentation using an improved icnet network with attention. Micromachines (Basel) 2022;13:1920.

[22] Park S, Erkinov H, Hasan MAM, Nam S-H, Kim Y-R, Shin J, et al. Periodontal disease classification with color teeth images using convolutional neural networks. Electronics (Basel) 2023;12:1518.

[23] Hossam A, Mohamed K, Tarek R, Elsayed A, Mostafa H, Selim S. Automated dental diagnosis using deep learning. 2021 16th International Conference on Computer Engineering and Systems (ICCES), IEEE; 2021, p. 1–5.

[24] Li W, Li L, Xu W, Guo Y, Xu M, Huang S, et al. Identification of gingival inflammation surface image features using intraoral scanning and deep learning. Int Dent J 2025.

[25] Chau RCW, Cheng ACC, Mao K, Thu KM, Ling Z, Tew IM, et al. External validation of an AI mHealth tool for gingivitis detection among older adults at daycare centers: a pilot study. Int Dent J 2025:(in press).
