# Supplementary material for: Deep Learning for Detecting Dental Plaque and Gingivitis From Oral Photographs: A Systematic Review
Source: Community Dent Oral Epidemiol. 2025 Jun 26;53(6):617–32. doi: 10.1111/cdoe.70001 (PMC12627268; doi:10.1111/cdoe.70001)
Supplement: Supplementary file 5 — Appendix S5 [file CDOE-53-617-s004.docx]

| **Appendix 5.** Risk of bias and applicability concerns based on QUADS-2 tool for included studies in chronological order | | | | | | | |
| --- | --- | --- | --- | --- | --- | --- | --- |
| **Study** | **Risk of Bias** | | | | **Applicability** | | |
|  | **Patient Selection** | **Index Test** | **Reference Standard** | **Flow and Timing** | **Patient Selection** | **Index Test** | **Reference Standard** |
| *Alalharith*  *(2020)* | unclear | low | low | low | unclear | low | low |
| *Carillo​*  *(2020)* | high | low | unclear | unclear | high | low | unclear |
| *Liang*  *(2020)* | unclear | low | high | low | low | low | unclear |
| *Li*  *(2020)* | low | low | low | low | low | low | low |
| *You*  *(2020)* | low | low | low | low | unclear | low | low |
| *Li*  *(2021)* | low | low | high | low | low | low | high |
| *Shang*  *(2021)* | unclear | low | unclear | low | unclear | low | unclear |
| *Li (wen)*  *(2021)* | low | low | low | low | low | low | low |
| *Kurt-Bayrakdar*  *(2022)* | low | low | low | low | low | low | low |
| *Andrade*  *(2022)* | unclear | low | low | low | unclear | low | low |
| *Li*  *(2022)* | low | low | low | low | low | low | low |
| *Chau*  *(2023)* | low | low | low | low | low | low | low |
| *Shi*  *(2023)* | low | low | low | low | low | low | low |
| *Li*  *(2024)* | low | low | low | low | low | low | low |
| *Yukesel*  *(2024)* | unclear | low | low | low | unclear | low | low |
| *Chen*  *(2024)* | low | low | low | low | unclear | low | low |
| *Wen*  *(2024)* | low | low | low | low | low | low | low |
| *Sobrinho*  *(2024)* | unclear | low | low | low | unclear | low | unclear |
| *Pedraza*  *(2024)* | low | low | unclear | low | low | low | unclear |
| *Nantakeeratipat*  *(2024)* | unclear | low | unclear | low | unclear | low | unclear |
| *Cheng*  *(2024)* | low | low | low | low | unclear | low | low |
| *Liu*  *(2024)* | low | low | unclear | low | low | low | low |
| *Ragadio*  (2024) | high | low | high | unclear | high | low | high |

*Abbreviations:*

QUADS-2: Quality Assessment of Diagnostic Accuracy Studies-2
